# Impact of socioeconomic status on chronic obstructive pulmonary disease prognosis: a national cohort study

**DOI:** 10.3389/fmed.2025.1584945

**Published:** 2025-06-04

**Authors:** Hyewon Lee, Jiyun Jung, Hee-Young Yoon

**Affiliations:** ^1^Department of Health Administration and Management, College of Medical Sciences, Soonchunhyang University, Asan, Republic of Korea; ^2^Department of Software Convergence, Soonchunhyang University Graduate School, Asan, Republic of Korea; ^3^Department of Biostatistics, Dongguk University College of Medicine, Gyeongju, Republic of Korea; ^4^Division of Pulmonary and Critical Care Medicine, Department of Internal Medicine, Soonchunhyang University Seoul Hospital, Seoul, Republic of Korea

**Keywords:** healthcare disparities, environmental exposure, survival rate, risk factors, longitudinal studies

## Abstract

**Background:**

Chronic obstructive pulmonary disease (COPD) is a major cause of global morbidity and mortality, with socioeconomic status (SES) playing a significant role in disease outcomes. While the impact of individual SES on COPD has been reported, the influence of both individual and neighborhood SES on clinical outcomes remains unclear. We aimed to evaluate the association between SES and COPD outcomes.

**Method:**

We conducted a retrospective cohort study using 2015–2018 data from the Korean National Health Insurance Service-National Sample Cohort, linked with census data. SES was assessed at both individual (income, insurance type) and neighborhood levels (residential area, elderly proportion, education level, gross regional domestic product, and total population density). Outcomes included overall mortality and hospitalization, which were evaluated using Cox proportional hazard models adjusted for demographic and air pollution.

**Results:**

Among 12,820 patients (mean age 63.5 years, 47.2% male), higher income was significantly associated with lower mortality risk (hazard ratio [HR] = 0.961, 95% confidence interval [CI] = 0.936–0.986) in the adjusted model. Suburban residence was associated with increased mortality risk (HR = 1.432, 95% CI = 1.089–1.884), while rural residence was not significant after adjustment. For hospitalization, higher income was also significantly associated with a lower risk (HR = 0.987, 95% CI = 0.979–0.995). Suburban (HR = 1.097, 95% CI = 1.013–1.187) and rural (HR = 1.138, 95% CI = 1.046–1.239) residence also remained significantly associated with increased hospitalization risk in the adjusted models. Additionally, a higher proportion of older adults (HR = 1.010, 95% CI = 1.004–1.016) and lower educational attainment (HR = 0.992, 95% CI = 0.989–0.995) were also significantly associated with hospitalization risk.

**Conclusion:**

These findings suggest that individual SES is associated with both mortality and hospitalization among patients with COPD, while neighborhood SES influences hospitalization but not mortality after adjustment.

## 1 Introduction

Chronic obstructive pulmonary disease (COPD) is a leading global cause of morbidity and mortality, particularly affecting older populations due to long-term exposure to harmful substances, such as tobacco smoke, air pollutants, and occupational hazards (1, 2). Despite medical advances, COPD remains a major health burden owing to its progressive nature, frequent exacerbations, and limited curative options (3), all of which reduce the quality of life and increase healthcare costs. While smoking is a primary risk factor, evidence shows that environmental factors, particularly socioeconomic status (SES) also significantly impact COPD outcomes.

SES is a well-documented determinant of health outcomes in various diseases (4), including COPD. Studies have consistently associated lower SES with poorer COPD outcomes, such as higher incidence (5–9), mortality (10–15), lung function (11), symptoms (16), hospitalizations (7, 13, 17), and acute exacerbations (16). Individuals with lower SES often face multiple disadvantages, including limited access to healthcare, higher comorbidity burdens, and environmental stressors, which can further worsen COPD outcome. Additionally, individuals with lower SES are more likely to reside in areas with higher air pollution levels, such as near industrial zones and high-traffic roads (18, 19), where prolonged exposure to air pollutants can exacerbate respiratory conditions and further increase their risk of adverse COPD outcomes (20, 21). While previous research has explored the impact of SES on COPD, few studies have examined both individual and neighborhood SES levels simultaneously. Therefore, we investigated the association between SES, at both individual and neighborhood levels, and COPD outcomes, including mortality and hospitalization.

## 2 Materials and methods

### 2.1 Data source

This retrospective study used data from the Korean National Health Insurance Service-National Sample Cohort (NHIS-NSC) from 2015 to 2018, supplemented by Korean census data from 2015. Korea’s universal healthcare system, managed by a single government insurer, covers nearly the entire population. The NHIS-NSC, established in 2002, includes a stratified random sample of 2.2% of the population, representing age, sex, region, insurance type, income, and medical expenses (22). It provides comprehensive data, including information on patient demographics, health behaviors, medical history, healthcare utilization, diagnoses, costs, and mortality, and prospectively tracks individuals from 2007 to 2019. Neighborhood characteristics were analyzed based on patient addresses.

This study was approved by the Institutional Review Board of the Soonchunhyang University Seoul Hospital (2023–06-008) and complied with the Declaration of Helsinki. Informed consent was waived due to the anonymized nature of the dataset. The data were accessed for research purposes on 15 October 2024. All patient data were anonymized before access, and the authors did not have access to any information that could identify individual participants during or after data collection.

### 2.2 Study population

**Figure 1:**
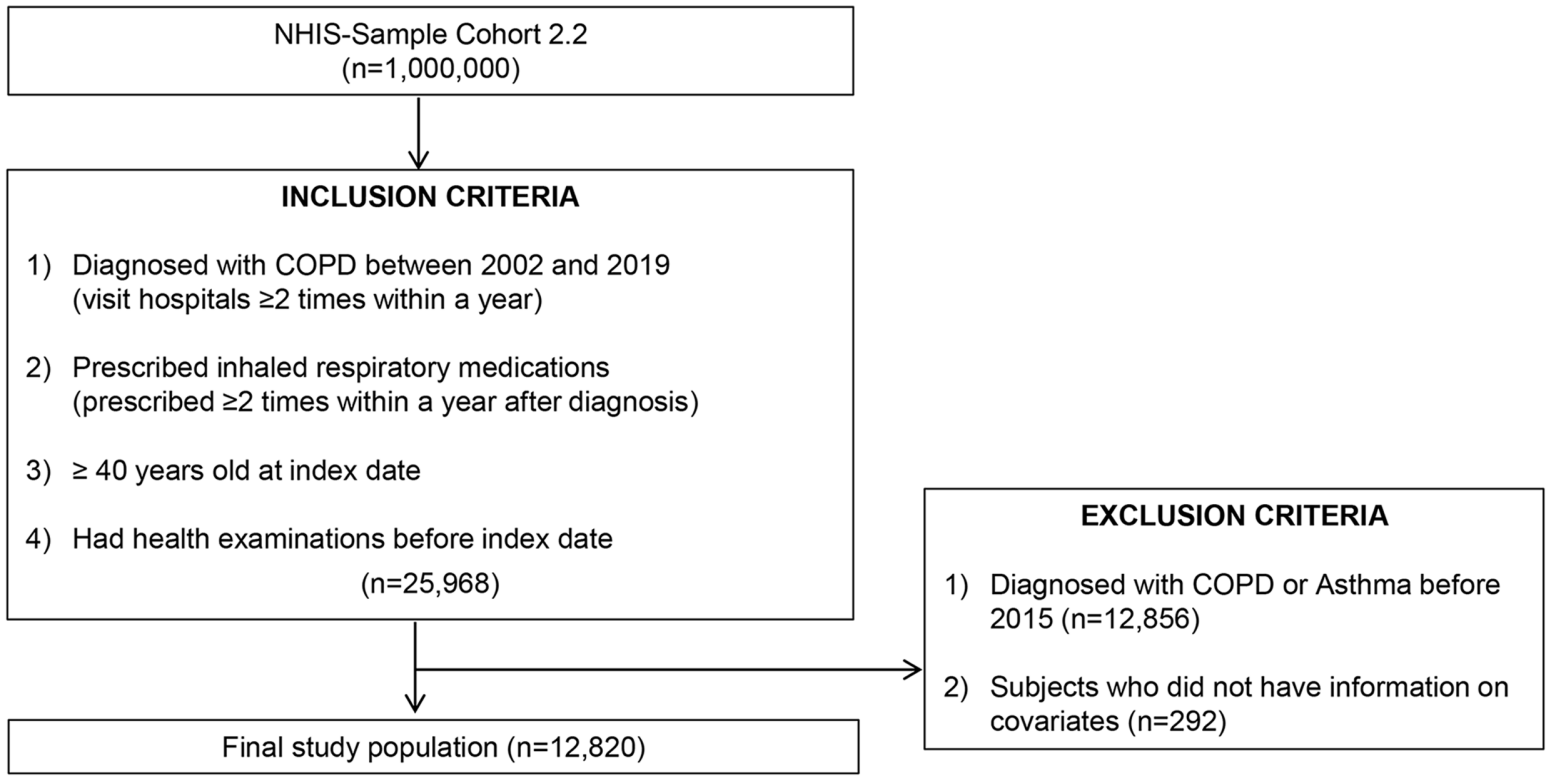
Enrolment of patients. NHIS, National Health Insurance Service; COPD, chronic obstructive pulmonary disease.

We screened 25,968 patients diagnosed with COPD between 2002 and 2018, identified by the International Classification of Diseases, Tenth Revision codes J41-J44 and J47, from the NHIS-NSC (Figure 1). The index date was defined as the first recorded date of COPD diagnosis. To be eligible, participants had to meet the following criteria: (1) at least two medical visits for COPD within 1 year of the initial diagnosis, (2) aged 40 years or older, due to the lower likelihood of COPD in younger individuals, (3) prescribed respiratory medications such as inhaled corticosteroids (ICS), long-acting muscarinic antagonists (LAMA), combined ICS/long-acting beta-agonists (LABA), LAMA/LABA, short-acting beta-agonists, xanthines, leukotriene receptor antagonists, or systemic steroids at least twice within 1 year of diagnosis, and (4) underwent national health examinations at the time of COPD diagnosis, ensuring the availability of major covariates, including smoking status. Patients diagnosed with COPD or asthma before 2015, when particulate matter with a diameter of ≤2.5 μm (PM2.5) data became available (*n* = 12,856), and those with missing key variables, such as residential address (*n* = 292), were excluded. Ultimately, 12,820 patients were included in the analysis.

### 2.3 Socioeconomic status exposure

We assessed two primary exposure variables, SES and long-term exposure to air pollution. SES was measured at both the individual and neighborhood levels. Individual SES, categorized by income level and insurance type (self-employed head, member, employee, employee-dependent, and medical aide), were obtained from the NHIS-NSC database. Neighborhood SES was assessed using resident area type (Metropolitan, Suburban, Rural), population aged ≥ 65 years (%), high school graduation rates, population density (2005 Census), and Gross Regional Domestic Product (GRDP) per capita data from 2010, the earliest available year at the district level (Korean Statistical Information Service, https://kosis.kr/index/index.do). These variables were determined based on the patient’s residential address in Si (city), Gun (county), and Gu (borough) administrative divisions to capture smaller regional variations.

### 2.4 Air pollutant exposure

Air pollution is a key factor influencing COPD outcomes and is correlated with SES, making it an essential adjustment variable in our analysis. To estimate long-term air pollution exposure in 2015, we used a validated prediction model based on data from AirKorea^1^ (23), which provides real-time air quality information across South Korea through 642 monitoring stations (Supplementary Figure 1). These stations measure six air pollutants— particulate matter with a diameter of 10 micrometers or less (PM10), and PM2.5, nitrogen dioxide (NO2), sulfur dioxide, ozone and carbon monoxide— hourly using consistent methods. Monitoring locations were selected based on the population density and regional characteristics for comprehensive coverage. To reduce exposure bias in the distribution of monitoring stations among regions, we applied Inverse Distance Weighting with squared-weighted interpolation to estimate pollutant concentrations. The detailed methods are provided Supplementary Table 1, and the predicted air pollutant distribution is shown in Supplementary Figure 2.

### 2.5 Outcome measures

The primary outcome was overall mortality from COPD diagnosis (index date) until death or censoring in December 2019. Secondary outcomes was time to first all-cause hospitalization.

### 2.6 Statistical analysis

Continuous variables are presented as mean ± standard deviation (SD) for normally distributed data and median (interquartile range [IQR]) otherwise. Normality was tested using the Shapiro–Wilk test. Categorical variables are summarized as counts and percentages. SES variables were analyzed both as a continuous variable and in quartiles (Q1–Q4) or subtypes for comparison.

Survival probabilities across SES groups were illustrated with Kaplan–Meier survival curves and compared using the log-rank test. These analyses were conducted using the ‘survival’ and ‘survminer’ packages in R. Cox proportional hazard models estimated hazard ratios (HRs) with 95% confidence intervals (CIs) to evaluate the associations between SES, air pollution exposure, and COPD outcomes. We tested the proportional hazards assumption using Schoenfeld residuals. Three models were developed: Model 1 represented the unadjusted analyses. Model 2 was adjusted for demographic and clinical covariates, including age, sex, smoking status, body mass index, year of COPD diagnosis, use of respiratory medications (ICS, LAMA, LABA, and systemic steroids), and the Charlson Comorbidity Index (CCI). Model 3 extended Model 2 by further adjusting for long-term exposure to air pollution.

All statistical analyses were performed using the SAS Enterprise Guide version 8.3 (SAS Institute Inc., Cary, NC, United States) and R Studio version 4.3.0 (RStudio Inc., Boston, MA, United States). Statistical packages used in R included ‘survival’, ‘survminer’, ‘dplyr’, and ‘ggplot2’. A two-sided *p*-value < 0.05 was considered statistically significant.

## 3 Results

### 3.1 Baseline demographics

The study included 12,820 patients with COPD, with a mean age of 63.5 years, 47.2% were male (Table 1). Most participants were non-smokers (64.8%), with 16.2% current smokers and 19.0% former smokers. The comorbidities included peptic ulcer disease (17.1%), diabetes without complications (15.1%), and cerebrovascular disease (7.8%). Mortality was 6.5%, with a median survival time of 2.60 years (IQR 1.48–3.23), and 49.0% experienced hospitalization, with a median time to first admission of 1.32 years (IQR 0.27–2.38). Socioeconomically, the income quartiles ranged from 19.3% in the lowest group to 30.0% in the highest group; 26.7% were employees, 41.2% were dependents, and 3.5% were on medical aid (Table 2). Most lived in metropolitan areas (56.5%), followed by suburban (27.9%), and rural (15.6%) areas. Annual average pollutant levels in 2015 were 25.6 μg/m^3^ for PM2.5, 47.1 μg/m^3^ for PM10, 24.1 ppb for NO2, 5.0 ppb for sulfur dioxide, 46.2 ppb for ozone, and 0.6 ppm for carbon monoxide.

**Table 1:** Baseline demographics.

Variables	*N* = 12,820
Age, years	63.5 ± 11.6
40–49	1,747 (13.6)
50–59	3,004 (23.4)
60–69	3,938 (30.7)
70–79	2,995 (23.4)
80 and above	1,136 (8.9)
Sex	
Male	6,053 (47.2)
Female	6,767 (52.8)
Smoking status	
Non-smoker	8,311 (64.8)
Former smoker	2,432 (19.0)
Current smoker	2,077 (16.2)
BMI, kg/m^2^	24.2 ± 3.4
Diagnosis year	
2015	2,395 (18.7)
2016	2,873 (22.4)
2017	2,858 (22.3)
2018	2,808 (21.9)
2019	1,886 (14.7)
Medications	
ICS only	1,036 (8.1)
ICS/LABA	665 (5.2)
LABA only	1,961 (15.3)
LABA/LAMA	679 (5.3)
LAMA only	396 (3.1)
SABA	2,505 (19.5)
Xanthine	3,860 (30.1)
LTRA	4,070 (31.8)
NAC	420 (3.3)
Systemic steroid	890 (6.9)
Oral	847 (6.6)
Intravenous	10 (0.1)
Comorbidity	
Myocardial infarction	121 (0.9)
Congestive heart failure	460 (3.6)
Peripheral vascular disease	1,083 (8.5)
Cerebrovascular disease	1,003 (7.8)
Dementia	164 (1.3)
Connective tissue disease	373 (2.9)
Peptic ulcer disease	2,197 (17.1)
Mild liver disease	1,328 (10.4)
Moderate or severe liver disease	12 (0.1)
Diabetes without complications	1,941 (15.1)
Diabetes with complications	690 (5.4)
Paraplegia and hemiplegia	84 (0.7)
Chronic kidney disease	165 (1.3)
Cancer, leukemia, lymphoma	750 (5.9)
Metastatic carcinoma	87 (0.7)
Lung comorbidity	
Asthma	2,732 (21.3)
ILD	42 (0.3)
Pulmonary thromboembolism	22 (0.2)
Cor pulmonale	4 (0.03)
CCI score	0.98 ± 1.4
0	6,219 (48.5)
1	3,517 (27.4)
2	1,594 (12.4)
≥3	1,490 (11.6)
Death	559 (4.4)
Time from diagnosis to death or censoring, years	2.60 (1.45–3.70)
Hospitalization	6,286 (49.0)
1 time	2,426 (18.9)
2 times	1,305 (10.2)
More than 3 times	2,555 (19.9)
Time from diagnosis to first hospitalization or censoring, years	1.32 (0.53–2.64)

BMI, Body Mass Index; ICS, inhaled corticosteroids; LABA, long-acting beta agonist; LAMA, long-acting muscarinic antagonist; SABA, short-acting beta-agonist; LTRA, leukotriene receptor antagonist; NAC, N-acetylcysteine; ILD, interstitial lung disease; CCI, Charlson Comorbidity index. Data are presented as numbers (%) or mean ± standard deviation, or median (interquartile range).

**Table 2:** Baseline individual and neighborhood socioeconomic status and air pollutants.

Variables	*N* = 12,820	Median (IQR)
Individual SES		
Incomes		
0–25%	2,470 (19.3)	
25–50%	2,608 (20.3)	
50–75%	3,898 (30.4)	
75–100%	3,844 (30.0)	
Health insurance type		
Self-employed head	2,379 (18.6)	
Self-employed member	1,286 (10.0)	
Employee	3,427 (26.7)	
Employee dependent	5,286 (41.2)	
Medical aid	442 (3.5)	
Neighborhood SES		
Individual resident type		
Metropolitan	7,247 (56.5)	
Sub-urban	3,577 (27.9)	
Rural	1,996 (15.6)	
Elderly population	8.2 (4.9)	6.2 (4.9–9.2)
0–20%	2,568 (20.0)	
20–50%	3,798 (29.6)	
50–80%	3,883 (30.3)	
80–100%	2,571 (20.1)	
High school graduates or higher	48.2 (9.6)	50.7 (44.4–54.2)
0–20%	2,580 (20.1)	
20–50%	3,783 (29.5)	
50–80%	3,890 (30.3)	
80–100%	2,567 (20.0)	
GRDP	5,877,920 (4,466,933)	4,623,213 (1,941,798–10,085,624)
0–20%	2,500 (19.5)	
20–50%	3,881 (30.3)	
50–80%	2,914 (22.7)	
80–100%	3,525 (27.5)	
Population density	270,230 (150,088)	272,715 (130,376–375,398)
0–20%	2,493 (19.5)	
20–50%	3,917 (30.6)	
50–80%	3,783 (29.5)	
80–100%	2,627 (20.5)	
Air pollution		
PM2.5, μg/m^3^	25.6 ± 1.9	25.5 (24.4–26.5)
PM10, μg/m^3^	47.1 ± 3.3	46.9 (45.3–49.3)
NO2, ppb	24.1 ± 5.5	22.2 (19.9–29.8)
SO2, ppb	5.0 ± 1.0	5.0 (4.5–5.5)
O3, ppb	46.2 ± 2.1	46.1 (44.8–47.8)
CO, ppm	0.6 ± 0.1	0.7 (0.6–0.7)

IQR, interquartile range; SES, Socioeconomic status; GRDP, Gross Regional Domestic Product; PM2.5, Particulate matter less than 2.5 microns; PM10, Particulate matter less than 10 microns; NO2, Nitrogen dioxide; SO2, Sulfur dioxide; O3, Ozone; CO, Carbon monoxide. Data are presented as number (%) or mean ± standard deviation.

### 3.2 Survival analysis between socioeconomic status and COPD prognosis

**Figure 2:**
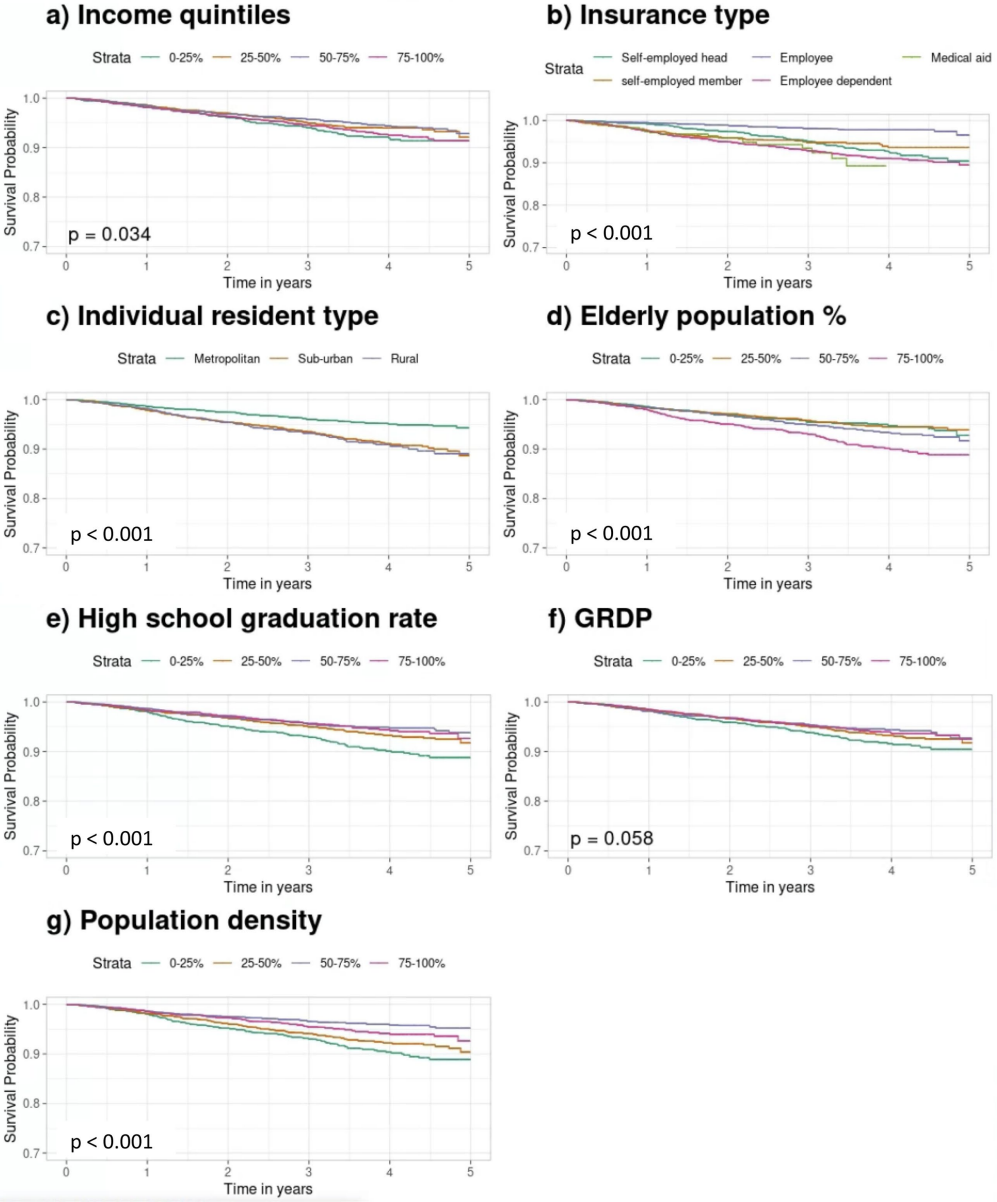
Kaplan–Meier survival curves for mortality curves according to individual and neighborhood SES quartiles. **(a)** Income quintiles. **(b)** Insurance type. **(c)** Individual resident type. **(d)** Older population. **(e)** High school graduation rates. **(f)** GRDP. **(g)** Population density. Kaplan–Meier survival curves show survival probabilities over time across SES groups. Differences were assessed using the log-rank test with vertical ticks indicating censored observations. SES, socioeconomic status; GRDP, Gross Regional Domestic Product.

In the Kaplan–Meier analysis, mortality significantly differed by individual income (Figures 2a,b; *p* = 0.037 overall), although pairwise income group differences were not significant (Supplementary Table 2). Mortality also varied by health insurance type, with employees and dependents having lower risks than the self-employed head (*p* < 0.001 and *p* = 0.005, respectively) and higher risks for medical aid recipients (*p* < 0.001). Suburban and rural residents had higher mortality than those in metropolitan areas (*p* < 0.001), although suburban-rural differences were not significant (*p* = 0.780) (Figure 2c). Higher proportions of older adults and lower education levels were associated with increased mortality (both *p* < 0.001), but differences among highly educated groups were not significant (*p* = 0.785) (Figures 2d,e). GRDP differences were marginally significant (*p* = 0.062) (Figure 2f), while higher population density correlated with greater mortality risk (*p* < 0.001) (Figure 2g).

**Figure 3:**
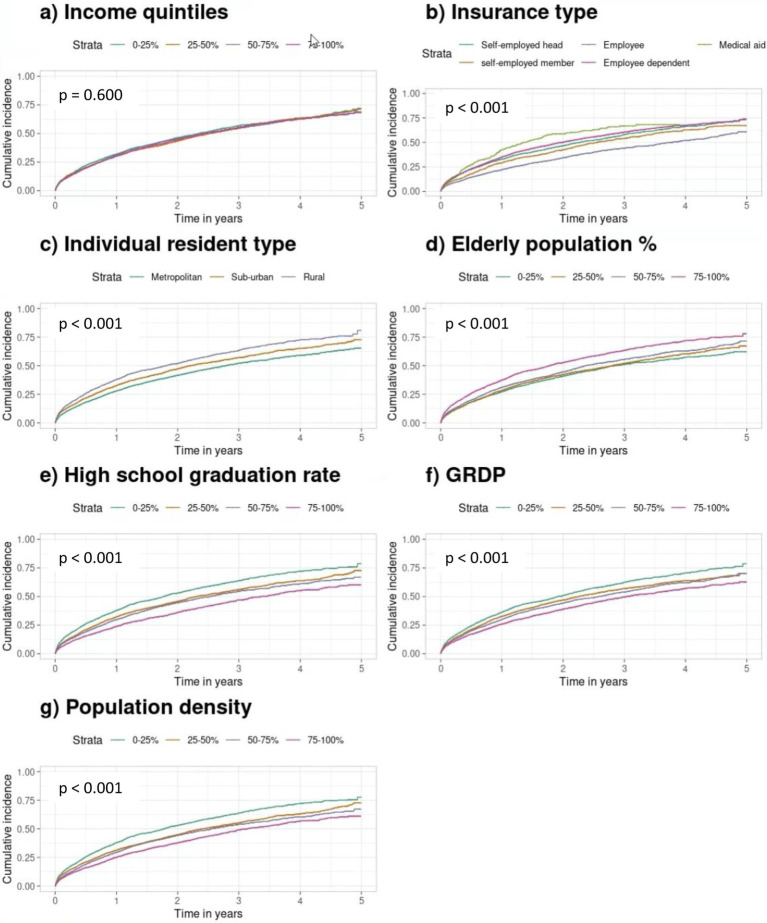
Kaplan–Meier survival curves for hospitalization curves according to individual and neighborhood SES quartiles. **(a)** Income quintiles. **(b)** Insurance type. **(c)** Individual resident type. **(d)** Older population. **(e)** High school graduation rate. **(f)** GRDP. **(g)** Population density. Kaplan–Meier survival curves show survival probabilities over time across SES groups. Differences were assessed using the log-rank test with vertical ticks indicating censored observations. SES, socioeconomic status; GRDP, Gross Regional Domestic Product.

Hospitalization outcomes also varied significantly across SES factors except for income quartiles (*p* = 0.660) (Figure 3a). Employees and dependents had lower risks than the self-employed head (*p* < 0.001 and *p* = 0.049), while medical aid recipients had the highest risk (*p* < 0.001) (Figure 3b). Suburban and rural residents showed increased risks compared to metropolitan residents (*p* < 0.001) (Figure 3c). Areas with higher proportions of older adults (80–100%) and lower education levels (0–20% high school graduates) had increased risks (both *p* < 0.001) (Figures 3d,e). Lower GRDP and higher population density were also associated with greater hospitalization risks (both *p* < 0.001) (Figures 3f,g).

### 3.3 Association between socioeconomic status and mortality

In the unadjusted model (Model 1), income was not significantly associated with mortality (Table 3). However, after adjusting for clinical factors (Model 2), lower income was associated with increased mortality risk (HR = 0.960, 95% CI = 0.935–0.985), a result that was held in the air pollution-adjusted model (Model 3, HR = 0.961, 95% CI = 0.936–0.986). Analysis by income quartiles showed patients in the 50–75% (Model 3: HR = 0.730, 95% CI = 0.57–0.932) and 75–100% (Model 3: HR = 0.694, 95% CI = 0.882–0.872) income groups had lower mortality risk than the 0–25% group. In the unadjusted models, employees had a lower mortality risk, while employee dependents had a higher risk. In adjusted models, only self-employed members showed a significantly increased risk (Model 3: HR = 2.165, 95% CI = 1.506–3.113), those receiving medical aid showed a marginally significant increase (Model 3: HR = 1.602, 95% CI = 0.987–2.598, *p* = 0.056).

**Table 3:** Association between socioeconomic status and mortality in patients with COPD.

	Model-1^a^		Model-2^b^		Model-3^C^	
	HR (95% CI)	*p*-value	HR (95% CI)	*p*-value	HR (95% CI)	*p*-value
Incomes	0.995 (0.967–1.023)	0.718	0.960 (0.935–0.985)	0.002	0.961 (0.936–0.986)	0.002
0–25%	Reference		Reference		Reference	
25–50%	0.784 (0.604–1.018)	0.067	0.813 (0.617–1.072)	0.143	0.804 (0.609–1.060)	0.122
50–75%	0.734 (0.577–0.933)	0.012	0.735 (0.575–0.939)	0.014	0.730 (0.571–0.932)	0.012
75–100%	0.928 (0.737–1.168)	0.523	0.688 (0.547–0.866)	0.001	0.694 (0.552–0.872)	0.002
Health insurance type						
Self-employed head	Reference		Reference		Reference	
Self-employed member	1.002 (0.724–1.386)	0.990	2.158 (1.499–3.107)	<0.001	2.165 (1.506–3.113)	<0.001
Employee	0.343 (0.245–0.480)	<0.001	0.970 (0.689–1.368)	0.864	0.995 (0.705–1.403)	0.976
Employee dependent	1.407 (1.128–1.753)	0.002	1.183 (0.941–1.487)	0.150	1.193 (0.948–1.501)	0.133
Medical aid	1.433 (0.899–2.285)	0.131	1.583 (0.977–2.565)	0.062	1.602 (0.987–2.598)	0.056
Individual resident type						
Metropolitan	Reference		Reference		Reference	
Sub-urban	1.802 (1.493–2.175)	<0.001	1.524 (1.252–1.855)	<0.001	1.432 (1.089–1.884)	0.010
Rural	1.863 (1.498–2.318)	<0.001	1.266 (1.011–1.587)	0.040	1.215 (0.905–1.633)	0.196
Older population	1.043 (1.029–1.058)	<0.001	1.012 (0.997–1.027)	0.131	1.002 (0.984–1.020)	0.852
0–20%	Reference		Reference		Reference	
20–50%	0.989 (0.760–1.286)	0.932	0.827 (0.626–1.093)	0.182	0.855 (0.646–1.131)	0.271
50–80%	1.178 (0.915–1.516)	0.205	0.903 (0.693–1.177)	0.451	0.829 (0.625–1.100)	0.194
80–100%	1.754 (1.363–2.258)	<0.001	1.058 (0.811–1.380)	0.676	0.917 (0.679–1.237)	0.570
High school graduates or higher	0.977 (0.970–0.985)	<0.001	0.992 (0.985–0.999)	0.044	0.999 (0.988–1.009)	0.784
0–20%	Reference		Reference		Reference	
20–50%	0.677 (0.545–0.842)	<0.001	0.908 (0.727–1.133)	0.394	0.928 (0.738–1.166)	0.519
50–80%	0.562 (0.449–0.705)	<0.001	0.814 (0.643–1.030)	0.087	0.958 (0.735–1.250)	0.753
80–100%	0.584 (0.453–0.751)	<0.001	0.805 (0.621–1.045)	0.103	0.984 (0.677–1.432)	0.934
GRDP						
0–20%	Reference		Reference		Reference	
20–50%	0.812 (0.648–1.018)	0.071	0.963 (0.764–1.214)	0.751	1.063 (0.840–1.345)	0.612
50–80%	0.750 (0.586–0.961)	0.023	1.080 (0.840–1.388)	0.548	1.231 (0.949–1.597)	0.118
80–100%	0.753 (0.594–0.953)	0.018	1.023 (0.803–1.303)	0.856	1.173 (0.854–1.609)	0.324
Total population density						
0–20%	Reference		Reference		Reference	
20–50%	0.810 (0.657–0.999)	0.049	1.070 (0.865–1.323)	0.533	1.062 (0.855–1.319)	0.589
50–80%	0.456 (0.357–0.582)	<0.001	0.681 (0.528–0.879)	0.003	0.727 (0.527–1.003)	0.052
80–100%	0.596 (0.463–0.767)	<0.001	0.801 (0.615–1.044)	0.100	0.835 (0.548–1.273)	0.402

COPD, Chronic obstructive pulmonary disease; HR, Hazard ratio; CI, Confidence interval; GRDP, Gross Regional Domestic Product.

^a^Model 1 presents unadjusted analyses.

^b^Model 2 was adjusted for demographic and clinical factors, including age, sex, smoking status, BMI, year of COPD diagnosis, use of respiratory medications (ICS, LAMA, LABA, and steroids), and CCI.

^c^Model 3 was adjusted for long-term exposure to air pollution.

For neighborhood SES, living in suburban or rural areas was associated with higher mortality in the unadjusted model. The association remained significant only for suburban areas (Model 3: HR = 1.432, 95% CI = 1.089–1.884). The proportion of older adult residents initially showed an association with mortality, which weakened in the air-pollution-adjusted model. Higher educational attainment in neighborhoods was associated with lower mortality in the unadjusted and clinically adjusted models (HR = 0.992, 95% CI = 0.985–0.999); however, it was not significant in Model 3. Similarly, both GRDP and population density were associated with lower mortality in the unadjusted model, although the significance diminished with adjustments, particularly for air pollution.

### 3.4 Association between socioeconomic status and hospitalization

In the unadjusted model, income was not significantly associated with hospitalization risk. However, after adjusting for clinical factors and air pollution, higher income was associated with a reduced hospitalization risk (Model 2: HR = 0.987, 95% CI = 0.979–0.995; Model 3: HR = 0.987, 95% CI = 0.979–0.995) (Table 4). Patients in the highest income quartile (75–100%) had a significantly lower hospitalization risk than those in the lowest quartile (0–25%) in the adjusted models (Model 2: HR = 0.903, 95% CI = 0.839–0.972; Model 3: HR = 0.901, 95% CI = 0.836–0.970). Employees had a lower hospitalization risk (Model 3: HR = 0.883, 95% CI = 0.814–0.959), while medical aid recipients consistently showed a higher risk, which remained significant after adjustments (Model 3: HR = 1.156, 95% CI = 1.000–1.337).

**Table 4:** Association between socioeconomic status and all-cause hospitalization in patients with COPD.

	Model-1^a^		Model-2^b^		Model-3^c^	
	HR (95% CI)	*p*-value	HR (95% CI)	*p*-value	HR (95% CI)	*p*-value
Incomes	0.995 (0.987–1.003)	0.188	0.987 (0.979–0.995)	0.001	0.987 (0.979–0.995)	0.001
0–25%	Reference		Reference		Reference	
25–50%	0.956 (0.884–1.034)	0.264	1.020 (0.941–1.104)	0.636	1.019 (0.940–1.104)	0.647
50–75%	0.964 (0.897–1.036)	0.321	0.996 (0.926–1.072)	0.917	0.993 (0.923–1.068)	0.846
75–100%	0.955 (0.888–1.027)	0.218	0.903 (0.839–0.972)	0.007	0.901 (0.836–0.970)	0.006
Health insurance type						
Self-employed head	Reference		Reference		Reference	
Self-employed member	0.884 (0.803–0.973)	0.012	0.993 (0.898–1.098)	0.890	0.992 (0.897–1.097)	0.877
Employee	0.668 (0.618–0.722)	<0.001	0.878 (0.809–0.953)	0.002	0.883 (0.814–0.959)	0.003
Employee dependent	1.069 (0.999–1.143)	0.052	0.982 (0.915–1.054)	0.611	0.973 (0.907–1.044)	0.445
Medical aid	1.301 (1.130–1.498)	<0.001	1.181 (1.023–1.365)	0.023	1.156 (1.000–1.337)	0.050
Individual resident type						
Metropolitan	Reference		Reference		Reference	
Sub-urban	1.189 (1.123–1.259)	<0.001	1.151 (1.086–1.219)	<0.001	1.097 (1.013–1.187)	0.023
Rural	1.415 (1.322–1.513)	<0.001	1.247 (1.163–1.336)	<0.001	1.138 (1.046–1.239)	0.003
Older population	1.028 (1.023–1.033)	<0.001	1.018 (1.013–1.023)	<0.001	1.010 (1.004–1.016)	0.001
0–20%	Reference		Reference		Reference	
20–50%	1.054 (0.978–1.135)	0.168	1.015 (0.942–1.094)	0.694	1.026 (0.951–1.107)	0.511
50–80%	1.153 (1.072–1.241)	<0.001	1.072 (0.996–1.155)	0.063	1.011 (0.936–1.092)	0.783
80–100%	1.455 (1.346–1.572)	<0.001	1.250 (1.154–1.353)	<0.001	1.109 (1.016–1.212)	0.021
High school graduates or higher	0.983 (0.981–0.986)	<0.001	0.988 (0.986–0.991)	<0.001	0.992 (0.989–0.995)	<0.001
0–20%	Reference		Reference		Reference	
20–50%	0.811 (0.757–0.868)	<0.001	0.883 (0.823–0.947)	<0.001	0.907 (0.844–0.974)	0.008
50–80%	0.755 (0.705–0.809)	<0.001	0.853 (0.795–0.916)	<0.001	0.883 (0.814–0.958)	0.003
80–100%	0.602 (0.557–0.652)	<0.001	0.675 (0.623–0.731)	<0.001	0.762 (0.684–0.849)	<0.001
GRDP						
0–20%	Reference		Reference		Reference	
20–50%	0.858 (0.801–0.919)	<0.001	0.917 (0.855–0.984)	0.016	0.963 (0.896–1.035)	0.307
50–80%	0.805 (0.748–0.866)	<0.001	0.889 (0.825–0.959)	0.002	0.930 (0.860–1.006)	0.071
80–100%	0.685 (0.637–0.736)	<0.001	0.751 (0.698–0.809)	<0.001	0.861 (0.783–0.946)	0.002
Total population density						
0–20%	Reference		Reference		Reference	
20–50%	0.799 (0.746–0.856)	<0.001	0.887 (0.826–0.951)	0.001	0.908 (0.845–0.975)	0.008
50–80%	0.749 (0.699–0.803)	<0.001	0.848 (0.789–0.911)	<0.001	0.873 (0.797–0.956)	0.003
80–100%	0.636 (0.589–0.688)	<0.001	0.707 (0.654–0.766)	<0.001	0.774 (0.683–0.876)	<0.001

COPD, Chronic obstructive pulmonary disease; HR, Hazard ratio; CI, Confidence interval; GRDP, Gross Regional Domestic Product.

^a^Model 1 presents unadjusted analyses.

^b^Model 2 was adjusted for demographic and clinical factors, including age, sex, smoking status, BMI, year of COPD diagnosis, use of respiratory medications (ICS, LAMA, LABA, and steroids), and CCI.

^c^Model 3 was adjusted for long-term exposure to air pollution.

In neighborhood SES analysis, suburban (Model 2: HR = 1.151, 95% CI = 1.086–1.219; Model 3: HR = 1.097, 95% CI = 1.013–1.187) and rural residents (Model 2: HR = 1.247, 95% CI = 1.163–1.336; Model 3: HR = 1.138, 95% CI = 1.046–1.239) showed higher hospitalization risks compared to metropolitan residents, although this was slightly reduced after adjusting for air pollution. A higher proportion of older patients was associated with increased hospitalization risk in Model 2 (HR = 1.018, 95% CI = 1.013–1.023) and a reduced effect size in Model 3 (HR = 1.010, 95% CI = 1.004–1.016). Higher educational attainment in neighborhoods was associated with a lower hospitalization risk, which was also attenuated in Model 3 (Model 2: HR = 0.988, 95% CI = 0.986–0.991; Model 3: HR = 0.992, 95% CI = 0.989–0.995). GRDP and population density in the higher quartiles were initially associated with a lower hospitalization risk; however, this significance decreased in the adjusted models, particularly in Model 3. Model 3 included the highest GRDP quartile (HR = 0.861, 95% CI = 0.783–0.946) and population density quartiles (50–80%: HR = 0.873, 95% CI = 0.797–0.956; 80–100%: HR = 0.774, 95% CI = 0.683–0.876) still showed reduced hospitalization risk.

## 4 Discussion

We used national claims data to examine the effects of individual and neighborhood SES on COPD outcomes. In our study, both individual and neighborhood SES were associated with COPD mortality and hospitalization. However, after adjusting for demographics and air pollution, the impact of neighborhood SES on mortality was reduced, while its association with individual SES, particularly income, remained significant. In contrast, both individual and neighborhood SES remained significantly associated with hospitalization. These findings highlight the distinct roles of individual and neighborhood SES in shaping COPD outcomes, with individual SES showing a stronger and more consistent association across both mortality and hospitalization.

We found a significant association between individual and neighborhood SES and COPD mortality, which is consistent with previous studies (10–15). A study using the Korean NHIS-NCS database (2002–2013) found that lower-income (HR = 1.39, 95% CI = 1.20–1.59) and middle-income patients (HR = 1.29, 95% CI = 1.15–1.44) with COPD (*n* = 9,275) had higher mortality risks compared to higher-income individuals (14). The risk was further elevated for those living in disadvantaged neighborhoods, while no significant income-related mortality difference was observed in advantaged neighborhoods (14). Similarly, in a USA Veteran Health Administration study of patients with COPD (*n* = 1,106,163), those in the highest deprivation quintile had higher mortality risk (adjusted odd ratio [OR] = 1.30, 95% CI = 1.28–1.32) than those in the lowest, while rural residents had a lower risk compared to urban residents (adjusted OR = 0.92, 95% CI = 0.89–0.95) (15). This trend suggests that individuals from lower socioeconomic backgrounds show greater COPD mortality risk owing to factors such as limited healthcare access, suboptimal living conditions, and higher occupational and environmental hazard exposure, all of which contribute to worse health outcomes. Our results align with this trend; however, after adjusting for clinical factors and air pollution, the association between neighborhood SES and mortality was attenuated, indicating that neighborhood SES may partly reflect other health determinants. Similar findings have been reported in studies on other respiratory conditions, such as a U.S. study showing that asthma-related emergency visit rates were higher in areas with larger Black and Latinx populations but decreased by 24 and 32%, respectively, after adjusting for air pollution (24). These findings highlight the complex interplay between SES and COPD outcomes and underscore the importance of considering both individual and community-level factors in understanding disease prognosis.

In our study, both individual and neighborhood SES showed a stronger influence on hospitalization than on mortality. This SES-related effect on hospitalization remained relatively stable even after adjusting for air pollution. A study in China (*n* = 39,054) observed that individuals with a higher SES (measured by education and income) showed a stronger association between PM10 exposure and COPD mortality than those with a lower SES (25). Unlike these studies, our findings suggest that hospitalization outcomes in patients with COPD may be more consistently influenced by immediate SES-related resources, such as healthcare access, rather than by long-term pollution exposure. In contrast, COPD mortality may be more sensitive to cumulative pollution exposure over time, which aligns with our observation that mortality is modulated to a greater extent by air pollution than by hospitalization.

Studies across regions have highlighted the complexity of interactions between SES and air pollution (26–28). A nationwide U.S. study found that higher PM2.5 exposure was strongly associated with increased mortality risk, particularly in low neighborhood SES census tracts, characterized by lower income, fewer college-educated residents, and higher proportions of Black and low-income populations in the general population (26). Similarly, do Nascimento and Gouveia (27) reported in São Paulo that low education and neighborhood SES were associated with higher non-accidental and respiratory mortality risks due to short-term PM10 and NO2 exposure, respectively. A Hong Kong study further supported this finding, showing that individuals with lower SES, especially those in public housing or blue-collar jobs, were more vulnerable to the acute effects of air pollution on mortality, although no significant interaction with educational level was found (28). These studies suggest that lower SES communities often face a disproportionate burden of pollution-related health risks due to factors such as closer proximity to pollution sources and weaker regulatory protections (29). While previous research has shown that air pollution can amplify SES-related health disparities, our findings demonstrated that SES had a consistent impact on COPD hospitalization across quartiles, even after adjusting for air pollution. This may indicate that factors associated with SES, such as healthcare access, baseline health status, and comorbidities, play a dominant role in hospitalization risk, which may not be as easily mitigated by environmental adjustments. Additionally, hospitalization is often driven by acute exacerbations and short-term health needs, which may be less sensitive to chronic environmental exposures compared to long-term mortality outcomes.

Our study had certain limitations. First, the retrospective NHIS-NSC design limits the causal inference between SES, air pollution, and COPD outcomes, despite adjustments for key demographic and clinical factors. Second, while we included individual and neighborhood-level SES indicators, the dataset lacked information on broader social determinants of health such as access to healthcare, dietary factors, and social support, which may also influence COPD outcomes. These unmeasured variables remain potential confounders. Third, because our study was based on a Korean cohort, generalisability may be limited owing to SES and pollution differences across countries. Future studies on diverse populations should provide broader insights. Fourth, as pulmonary function data were not available in the dataset, we were unable to perform analyses stratified by COPD severity. However, to account for patients’ overall health status, we included the CCI as an adjustment variable in all multivariable models. Despite these limitations, our study underscores the critical role of socioeconomic and environmental factors in COPD management and policies.

## 5 Conclusion

In conclusion, this study demonstrated that SES is consistently associated with COPD hospitalization, while its impact on mortality was reduced after adjusting for air pollution. These findings underscore the importance of addressing both socioeconomic and environmental factors in public health strategies. Future research should further investigate the interaction between SES and air pollution using longitudinal and individual-level exposure data to develop targeted interventions for COPD management.

## Data Availability

The raw data supporting the conclusions of this article will be made available by the authors, without undue reservation.

## Footnotes

^1^ www.airkorea.or.kr
