# Supplementary material for: Impact of socioeconomic status on chronic obstructive pulmonary disease prognosis: a national cohort study
Source: Front Med (Lausanne). 2025 Jun 4;12:1584945. doi: 10.3389/fmed.2025.1584945 (PMC12173868; doi:10.3389/fmed.2025.1584945)
Supplement: Supplementary file 1 [file Table_1.docx]

Supplementary Material


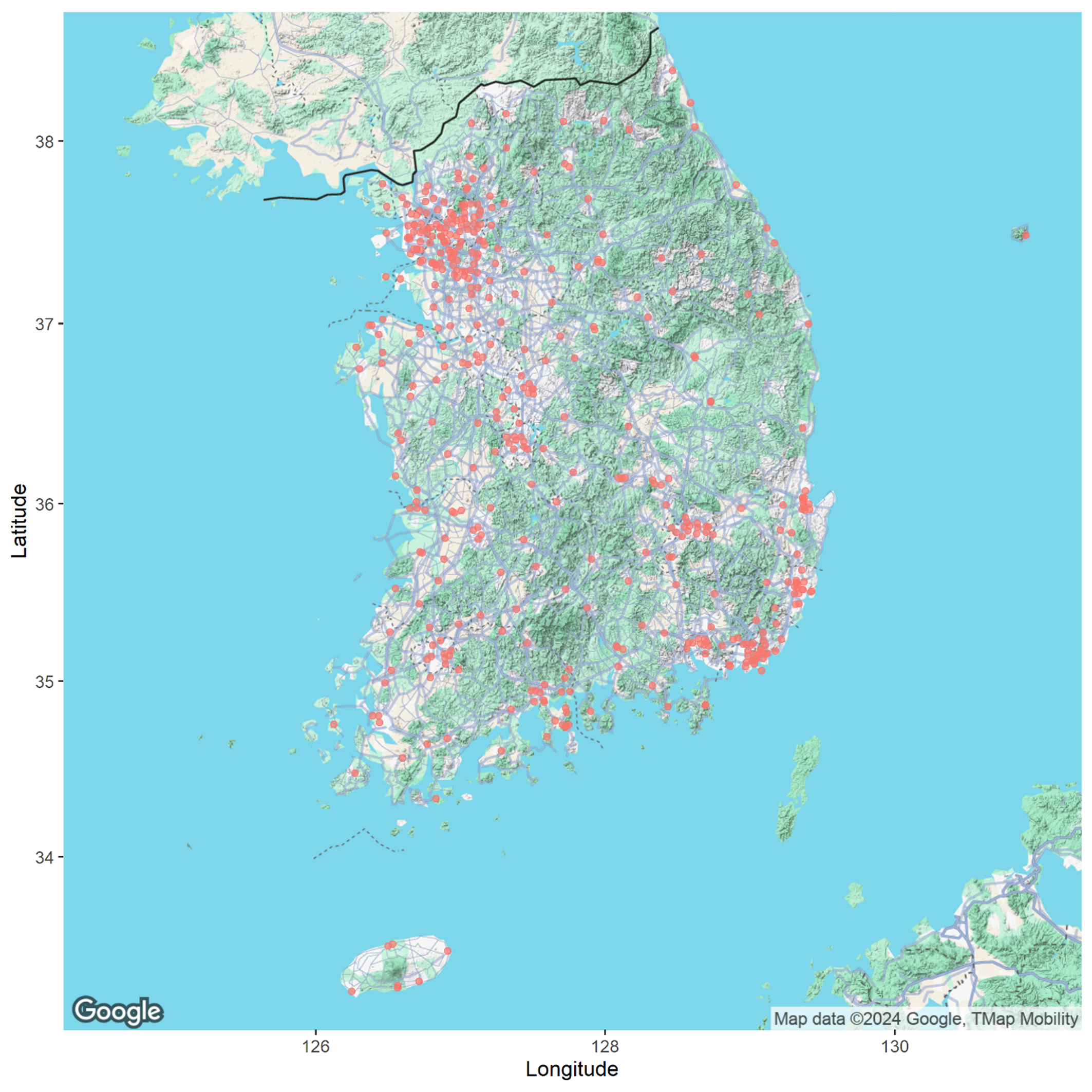


**Supplementary Figure 1.** **Geographical distribution of air quality monitoring stations in South Korea.** This map displays the distribution of air quality monitoring stations across South Korea, represented by red circles. These stations are strategically located to provide coverage across populated and industrial areas for comprehensive air pollution monitoring.


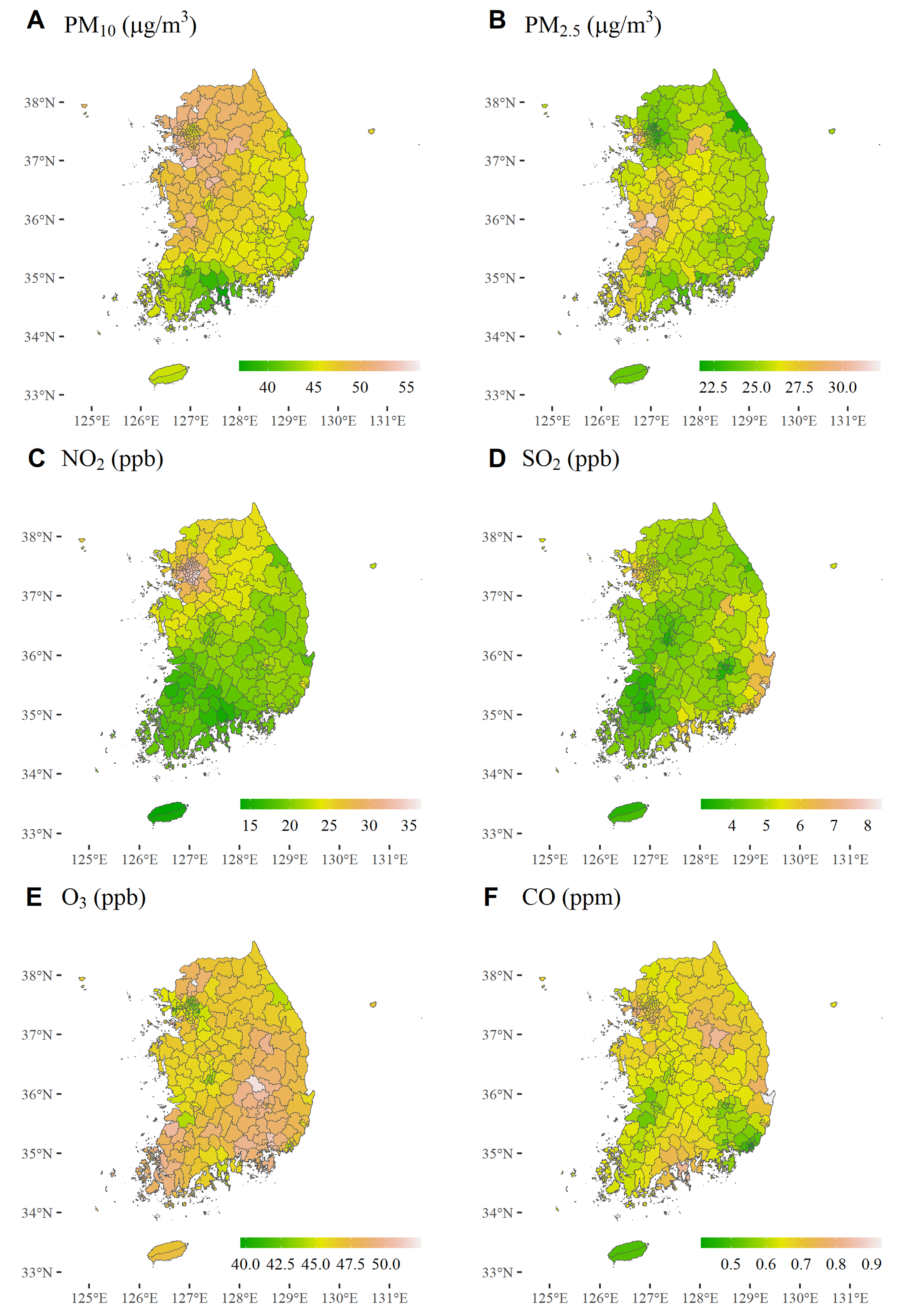


**Supplementary Figure 2.** **Geographical distribution of predicted air pollutant levels in South Korea.** These maps depict the predicted spatial distribution of various air pollutants across South Korea in 2015. Data include concentrations for PM2.5 (A), PM10 (B), NO2 (C), SO2 (D), O3 (E), and CO (F). Color gradients represent pollution levels by region, with higher intensity in red indicating areas with elevated pollutant concentrations and green indicating lower concentrations. PM10, Particulate matter less than 10 microns; PM2.5, Particulate matter less than 2.5 microns; NO2, Nitrogen dioxide; SO2, Sulfur dioxide; O3, Ozone; CO, Carbon monoxide.

| **Detailed Methodology** |
| --- |
| To estimate long-term air pollution exposure for the year 2015, we used a previously validated prediction model developed from data provided by the AirKorea website (www.airkorea.or.kr). AirKorea, under the National Ambient Air Information System managed by the Korean Ministry of Environment, has been collecting real-time air quality data nationwide since 2004. The network comprises 642 monitoring stations across 162 cities and counties in South Korea, measuring six air pollutants: particulate matter with a diameter of 10 micrometers or less (PM_10_), particulate matter with a diameter of 2.5 micrometers or less (PM_2.5_), nitrogen dioxide (NO_2_), sulfur dioxide (SO_2_), ozone (O_3_), and carbon monoxide (CO). Each station conducts measurements hourly using standardized and consistent methodologies. The stations are strategically placed, considering population density, urban development, and regional characteristics, to maximize spatial coverage.  However, due to uneven distribution and the absence of monitoring stations in some areas, we developed a prediction model to estimate air pollutant concentrations at the grid unit. The model employed spatial interpolation techniques, specifically inverse distance weighting (IDW), to improve the precision of exposure estimates. IDW calculates pollutant concentrations at grid considering the squared inverse distance between monitoring stations and grid. To allocate the exposure at geographical unit, we averaged grided exposure according to geographic scope. Each participant's exposure was matched according to residential address. |

**Supplementary Table 1. Details of the method used for predicting air pollutant concentrations.**

|  | post-hoc p-value using BH(Benjamini-Hochberg Procedure) | | | | | | | | | | |  |
| --- | --- | --- | --- | --- | --- | --- | --- | --- | --- | --- | --- | --- |
|  | Mortality | | | | | | Hospitalization | | | | |  |
| **Incomes** |  | **1** | **2** | **3** | **4** |  | | **1** | **2** | **3** | **4** | |
| 0-25% | **1** |  |  |  |  | **1** | |  |  |  |  | |
| 25-50% | **2** | 0.132 |  |  |  | **2** | | 0.66 |  |  |  | |
| 50-75% | **3** | 0.07 | 0.606 |  |  | **3** | | 0.66 | 0.99 |  |  | |
| 75-100% | **4** | 0.606 | 0.248 | 0.096 |  | **4** | | 0.66 | 0.99 | 0.99 |  | |
| **Health insurance type** |  |  |  |  |  |  | |  |  |  |  | |
| Self-employed head | **1** |  |  |  |  | **1** | |  |  |  |  | |
| Self-employed member | **2** | 0.991 |  |  |  | **2** | | 0.013 |  |  |  | |
| Employee | **3** | <.001 | <.001 |  |  | **3** | | <.001 | <.001 |  |  | |
| Employee dependent | **4** | 0.005 | 0.031 | <.001 |  | **4** | | 0.049 | <.001 | <.001 |  | |
| Medical aid | **5** | 0.069 | 0.306 | <.001 | 0.991 | **5** | | <.001 | <.001 | <.001 | 0.008 | |
| **Individual resident type** |  |  |  |  |  |  | |  |  |  |  | |
| Metropolitan | **1** |  |  |  |  | **1** | |  |  |  |  | |
| Sub-urban | **2** | <.001 |  |  |  | **2** | | <.001 |  |  |  | |
| Rural | **3** | <.001 | 0.78 |  |  | **3** | | <.001 | <.001 |  |  | |
| **Elderly population** |  |  |  |  |  |  | |  |  |  |  | |
| 0-20% | **1** |  |  |  |  | **1** | |  |  |  |  | |
| 20-50% | **2** | 0.917 |  |  |  | **2** | | 0.166 |  |  |  | |
| 50-80% | **3** | 0.251 | 0.197 |  |  | **3** | | <.001 | 0.008 |  |  | |
| 80-100% | **4** | <.001 | <.001 | <.001 |  | **4** | | <.001 | <.001 | <.001 |  | |
| **High school graduates or higher** |  |  |  |  |  |  | |  |  |  |  | |
| 0-20% | **1** |  |  |  |  | **1** | |  |  |  |  | |
| 20-50% | **2** | <.001 |  |  |  | **2** | | <.001 |  |  |  | |
| 50-80% | **3** | <.001 | 0.166 |  |  | **3** | | <.001 | 0.029 |  |  | |
| 80-100% | **4** | <.001 | 0.3 | 0.785 |  | **4** | | <.001 | <.001 | <.001 |  | |
| **GRDP** |  |  |  |  |  |  | |  |  |  |  | |
| 0-20% | **1** |  |  |  |  | **1** | |  |  |  |  | |
| 20-50% | **2** | 0.146 |  |  |  | **2** | | <.001 |  |  |  | |
| 50-80% | **3** | 0.066 | 0.615 |  |  | **3** | | <.001 | 0.066 |  |  | |
| 80-100% | **4** | 0.066 | 0.615 | 0.986 |  | **4** | | <.001 | <.001 | <.001 |  | |
| **Total population density** |  |  |  |  |  |  | |  |  |  |  | |
| 0-20% | **1** |  |  |  |  | **1** | |  |  |  |  | |
| 20-50% | **2** | 0.055 |  |  |  | **2** | | <.001 |  |  |  | |
| 50-80% | **3** | <.001 | <.001 |  |  | **3** | | <.001 | 0.048 |  |  | |
| 80-100% | **4** | <.001 | 0.02 | 0.055 |  | **4** | | <.001 | <.001 | <.001 |  | |

**Supplementary Table 2.** Post-hoc p-values for mortality and hospitalization by socioeconomic and demographic factors. Post-hoc p-values were calculated using the Benjamini-Hochberg procedure to adjust for multiple comparisons. GRDP, Gross Regional Domestic Product.
